# Nomogram-Based Prediction of the Risk of Diabetic Retinopathy: A Retrospective Study

**DOI:** 10.1155/2020/7261047

**Published:** 2020-06-07

**Authors:** Ruohui Mo, Rong Shi, Yuhong Hu, Fan Hu

**Affiliations:** School of Public Health, Shanghai University of Traditional Chinese Medicine, 201203, China

## Abstract

**Objectives:**

This study is aimed at developing a risk nomogram of diabetic retinopathy (DR) in a Chinese population with type 2 diabetes mellitus (T2DM).

**Methods:**

A questionnaire survey, biochemical indicator examination, and physical examination were performed on 4170 T2DM patients, and the collected data were used to evaluate the DR risk in T2DM patients. By operating R software, firstly, the least absolute shrinkage and selection operator (LASSO) regression analysis was used to optimize variable selection by running cyclic coordinate descent with 10 times *K* cross-validation. Secondly, multivariable logistic regression analysis was applied to build a predicting model introducing the predictors selected from the LASSO regression analysis. The nomogram was developed based on the selected variables visually. Thirdly, calibration plot, receiver operating characteristic (ROC) curve, and decision curve analysis were used to validate the model, and further assessment was running by external validation.

**Results:**

Seven predictors were selected by LASSO from 19 variables, including age, course of disease, postprandial blood glucose (PBG), glycosylated haemoglobin A1c (HbA1c), uric creatinine (UCR), urinary microalbumin (UMA), and systolic blood pressure (SBP). The model built by these 7 predictors displayed medium prediction ability with the area under the ROC curve of 0.700 in the training set and 0.715 in the validation set. The decision curve analysis curve showed that the nomogram could be applied clinically if the risk threshold is between 21% and 57% and 21%-51% in external validation.

**Conclusion:**

Introducing age, course of disease, PBG, HbA1c, UCR, UMA, and SBP, the risk nomogram is useful for prediction of DR risk in T2DM individuals.

## 1. Introduction

It is well-known that diabetes mellitus is a group of metabolic diseases characterized by hyperglycemia. Meanwhile, it is a major risk factor for microvascular disease. Nowadays, diabetes mellitus has become one of the world's fastest-growing chronic diseases. Since 2000, the International Diabetes Federation has reported the national, regional, and global occurrence of diabetes mellitus [1]. In 2009, it was estimated that 285 million people had diabetes mellitus [2], increasing to 366 million in 2011 [3], 382 million in 2013 [4], 415 million in 2015 [5], and 425 million in 2017 [6]. The newest report from the International Diabetes Federation showed that in 2019, there were 463 million (age 20~79 years) people living with diabetes mellitus worldwide and the number was expected to increase to 578 million by 2030 and to 700 million by 2045 [7]. With the increasing prevalence of diabetes mellitus and the aging of the population, the prevalence of diabetic microvascular complications such as diabetic retinopathy (DR) is likely to increase in parallel [8].

DR is a sight-threatening microvasculature impairment that seriously impacts the lives of diabetic patients. The global prevalence of DR, for the period 2015 to 2019, was 27.0% for any DR comprising of 25.2% nonproliferative DR and 1.4% proliferative DR [9]. With the increasing number of people with diabetes mellitus, the number of DR and vision-threatening DR, which includes severe nonproliferative DR, proliferative DR, and diabetic macular edema, has been estimated to rise to 191.0 million and 56.3 million, respectively, by 2030 [5].

The burden of an increasing population of diabetes mellitus will be more and more heavy based on the above reports. Since it has become an unstoppable trend, it is extremely necessary to take prevention seriously to decrease the incidence of associated complications. Therefore, our study tended to develop a risk nomogram for the prediction of DR.

## 2. Materials and Methods

### 2.1. Study Design and Setting

This study was based on a cross-sectional study conducted in 6 different communities from Yangpu District and Pudong New District in Shanghai from September 2015 to December 2018. A randomized stratified multiple-stage sampling method was used to select a representative sample. At first, Yangpu District belonging to urban areas and Pudong New District belonging to suburban areas were randomly selected. Then, 6 communities were randomly chosen from the two districts, including Huamu Community, Jinyang Community, and Sanlin Community in Yangpu District and Yinhang Community, Siping Community, and Daqiao Community in Pudong New District. The cross-sectional study is aimed at investigating the situation about type 2 diabetes mellitus (T2DM) from community grassroots in Shanghai by cooperating with the community health centers who were responsible for the management of chronic diseases and had a health registration system for the residents with diabetes within its range of services. The study was approved by the Institutional Review Board of Shanghai Jiao Tong University School of Medicine and was performed abiding by the principles of the Declaration of Helsinki of 1975 which was revised in 2008. Therefore, based on the data from the cross-sectional study, this study was about to discover the risk factors associated with DR and develop a predictive model to present the influence of those risk factors visually and quantitatively.

### 2.2. Study Participants

4170 T2DM patients were included in this study in total. And the criteria for inclusion were as follows: (1) T2DM patients own a registered regional household or live in the community for more than 6 months and have been included in community health registration system; (2) T2DM patients must be at least 18 years old; (3) T2DM was diagnosed according to the international diagnostic criteria and classification declared by the WHO in 1990 which defined diabetes according to a fasting plasma glucose concentration of 7.0 mmol/L (126 mg/dL) or higher, or 2 h postglucose load venous plasma glucose of 11.1 mmol/L (200 mg/dL) or higher, or random plasma glucose concentration of 11.1 mmol/L (200 mg/dL) or higher; and (4) all participating patients were provided written informed consent and willing to participate in the study.

### 2.3. Data Collection

**Figure 1:**
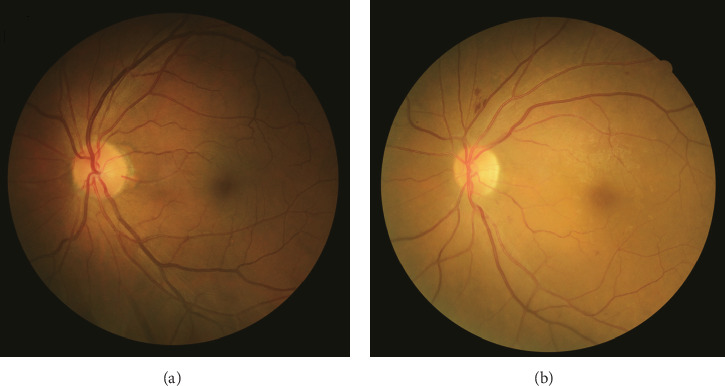
The normal fundus characteristics of T2DM patients (a) and the abnormal fundus characteristics of DR patients (b).

All subjects received questionnaire survey, biochemical indicator examination, physical examination, and fundus examination. The questionnaire survey was used to receive the basic information of the subjects, including age, gender, and course of disease. Biochemical indicator examination was conducted in the morning when all subjects were on fasting state and postprandial state, so fasting blood, 2 h postprandial blood, and morning urine were collected. It focused on the examination of fasting blood glucose (FBG), postprandial blood glucose (PBG), glycosylated haemoglobin A1c (HbA1c), total cholesterol (TC), total triglycerides (TG), low-density lipoprotein (LDL), high-density lipoprotein (HDL), serum creatinine (SCR), blood urea nitrogen (BUN), uric acid (UA), urine creatinine (UCR), and urinary microalbumin (UMA). Physical examination was focused on the measurement of blood pressure, height, weight, waist circumference, hip circumference, and the body mass index (BMI), and waist-to-hip ratio (WHR) was calculated. To assess the severity of DR, fundus examination was performed for all subjects by using a nonmydriatic fundus camera (CR-2 AF, Canon Inc., Tokyo, Japan) to get their digital, color, and nonstereoscopic retinal photographs. 45° digital retinal photographs were captured on the posterior pole of each eye (Figures 1(a) and 1(b)). The photographs were saved in JPG format (2592 × 1728 pixels) and then independently graded by a professionally trained ophthalmologist who was aware of the clinical details. After fundus screening, those who had fundus problem would be suggested to have a review in the department of ophthalmology at Shanghai University of Traditional Chinese Medicine Affiliated Hospital within one week. The result of the review would be followed up and determine whether DR was diagnosed. The severity of DR was categorized into five stages according to the International Clinical Diabetic Retinopathy Disease Severity Scale (2002) as follows: no DR, mild nonproliferative DR, moderate nonproliferative DR, severe nonproliferative DR, and proliferative DR [10]. The grade of DR in each individual was classified on the basis of the worse or only gradable eye.

### 2.4. Statistical Analysis

Statistical analysis was performed using the R software (version 3.6.1; https://www.R-project.org). 4170 T2DM patients in the study were randomly divided into a training set with 3130 participants and a validation set with 1040 participants for external validation conformed to the theoretical ratio of 3 : 1 [11]. The least absolute shrinkage and selection operator (LASSO) regression analysis is a shrinkage and variable selection method for linear regression models. In order to obtain the subset of predictors, the LASSO regression analysis minimizes prediction error for a quantitative response variable by imposing a constraint on the model parameters that cause regression coefficients for some variables to shrink toward zero. Variables with a regression coefficient equal to zero after the shrinkage process are excluded from the model while variables with nonzero regression coefficient are most strongly associated with the response variable. Based on the type measure of -2log-likelihood and binomial family, the LASSO regression analysis running in R software runs 10 times *K* cross-validation for centralization and normalization of included variables and then picks the best lambda value. “Lambda.lse” gives a model with good performance but the least number of independent variables. So the LASSO method was used to analyze the data in the training set to select the optimal predictors in the present risk factors including age, gender, course of disease, FBG, PBG, HbA1c, TC, TG, LDL, HDL, SCR, BUN, UA, UCR, UMA, systolic blood pressure (SBP), diastolic blood pressure (DBP), BMI, and WHR. Then, multivariable logistic regression analysis was used to build a predicting model by introducing the feature selected in the LASSO regression model [12]. The features were considered as odds ratio and *P* value with 95% confidence interval (CI). The statistical significance levels were all two-sided. Introducing all the selected features and analyzing statistical significance levels of the features, the statistically significant predictors were applied to develop a predicting model for DR risk. In our study, all the selected features had statistical significance and were applied to develop the nomogram prediction models.

Further, several kinds of validation methods were used to estimate the accuracy of the risk prediction model by using the data of the training set and the validation set, respectively. The area under the receiver operating characteristic (ROC) curve was used to provide good discrimination for the quality of the risk nomogram to separate true positives from false positives [13]. The calibration curve was used to evaluate the calibration of the DR risk nomogram, accompanied by the Hosmer-Lemeshow test. The decision curve analysis was used to determine the clinical practicability of nomograms based on the net benefit under different threshold probabilities in the T2DM cohort [14].

## 3. Results

### 3.1. Participant Characteristics

A total of 4170 T2DM patients including 2412 females and 1758 males [15, 16] in our study were composed of 3281 T2DM patients without DR and 889 T2DM patients with DR. After random sampling in a ratio of 3 : 1, 3130 and 1040 T2DM patients were included in the training set and validation set, respectively. All patients completed the related examination, and the data of patients in the two sets is given in Table 1.

### 3.2. Independent Risk Factors in the Training Set

**Figure 2:**
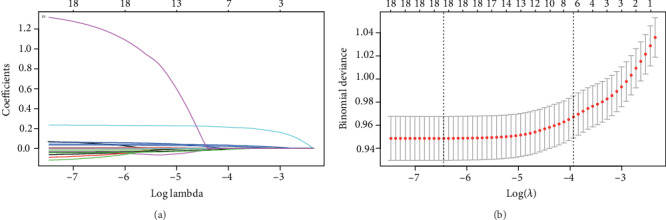
Variable selection by LASSO binary logistic regression model. A coefficient profile plot was produced against the log(lambda) sequence (a). Seven variables with nonzero coefficients were selected by optimal lambda. By verifying the optimal parameter (lambda) in the LASSO model, the partial likelihood deviance (binomial deviance) curve was plotted versus log(lambda) and dotted vertical lines were drawn based on 1 standard error criteria (b).

In all 19 associated characteristic variables, 7 potential predictors were selected on the basis of the data from the training set (Figures 2(a) and 2(b)) and were with nonzero coefficients in the LASSO regression model. These predictors included age, course of disease, PBG, HbA1c, UCR, UMA, and SBP.

### 3.3. Prediction Model Development

**Figure 3:**
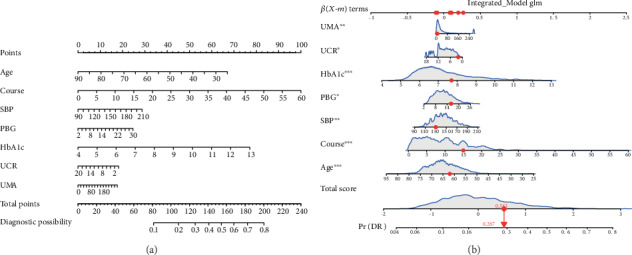
Development of the risk nomogram (a) and the dynamic nomogram for an example (b). The DR risk nomogram was developed with the predictors including age, course of disease, PBG, HbA1c, UCR, UMA, and SBP.

The results of the logistic regression analysis among age, course of disease, PBG, HbA1c, UCR, UMA, and SBP are given in Table 2. All these 7 predictors showed significant statistical differences. So introducing the above 7 independent predictors, a DR risk nomogram was developed and is presented in Figure 3(a). Meanwhile, the APP developed by using R language with the shiny package was applied in this study (URL: https://doctorfanhu.shinyapps.io/DR_DynNomapp/), which was mainly used to help clinical prediction and promote individualized evaluation. As an example to better explain the nomogram model, if the subject of T2DM is age of 62, course of 15 years, PBG of 16.0 mmol/l, HbA1c of 7.7%, UCR of 2.04 mmol/L, UMA of 5.00 mg/L, and SBP of 130 mmHg, the probability of DR is estimated to be 28.4% (Figure 3(b)).

### 3.4. Prediction Model Validation

**Figure 4:**
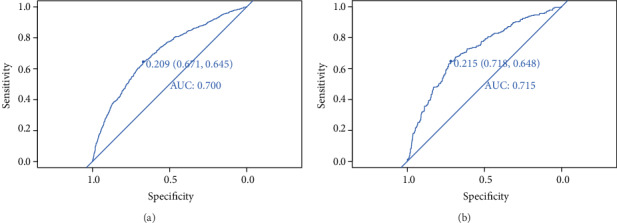
ROC validation of the DR risk nomogram prediction. The *y*-axis meant the true-positive rate of the risk prediction. The *x*-axis meant the false-positive rate of the risk prediction. The blue line represented the performance of the nomogram.  Figure 5(a) from the training set and Figure 5(b) from the validation set.

**Figure 5:**
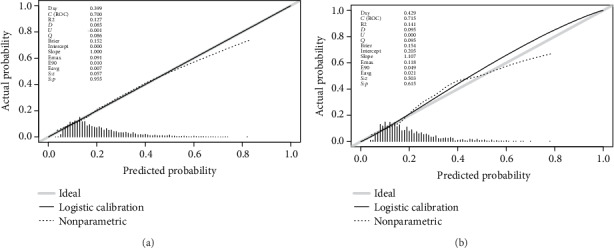
Calibration curves of the DR risk nomogram prediction. The *y*-axis meant the actual diagnosed DR. The *x*-axis meant the predicted risk of DR. The diagonal dotted line meant a perfect prediction by an ideal model. The solid line represented the performance of the training set (a) and validation set (b), which indicated that a closer fit to the diagonal dotted line represented a better prediction.

For the predictive model, the pooled area under the ROC curve of the nomogram was 0.700 in the training set and 0.715 in the validation set (Figures 4(a) and 4(b)), which indicated moderately good performance. The calibration curve of the nomogram to predict the DR risk in T2DM patients also showed good agreement (Figures 5(a) and 5(b)). To sum up the results from the above validation, the nomogram of the model had good prediction ability.

**Figure 6:**
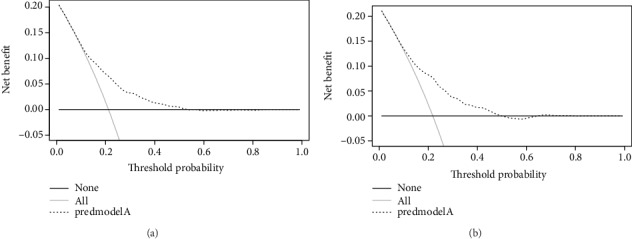
Decision curve analysis for the DR risk nomogram. The *y*-axis measured the net benefit. The thick solid line represented the assumption that all patients had no DR. The thin solid line represented the assumption that all patients had DR. The dotted line represented the risk nomogram. (a) From the training set and (b) from the validation set.

The decision curve showed that it would be more accurate to use this nomogram in the current study to predict the risk of DR when the risk threshold probability was between 21% and 57%, and in the validation set, it was between 21% and 51% (Figures 6(a) and 6(b)). Within this range, the net benefit was comparable with several overlaps, according to the nomogram.

## 4. Discussion

Nomograms are considered reliable and pragmatic prediction tools, with the ability to generate an individual probability of a clinical event by integrating diverse prognostic and determinant variables [ [17]], and incorporate multiple significant prognostic factors to quantify individual risk [18]. Nomograms meet our desire for biologically and clinically integrated models and fulfill our drive towards personalized medicine [17]. Depending on user-friendly digital interfaces, it more easily understood prognoses to aid better clinical decision making [12].

In this study, about 21.3% of the T2DM patients have been complicated with DR. In the risk factor analysis, age, course of disease, PBG, HbA1c, UCR, UMA, and SBP are associated with the risk of DR in T2DM patients. Based on that, we built and validated a novel prediction tool for the risk of DR among T2DM patients using these 7 available variables. This predictive model suggested that younger age and longer course of disease, higher PBG and HbA1c, lower UCR and higher UMA, and higher SBP were the key individual factors that determined the risk of DR for T2DM patients. By introducing basic information and biochemical and physical examination indicators into the DR risk nomogram, it was beneficial and convenient for the T2DM individualized prediction of risk of DR. This study provided a relatively accurate prediction tool of risk of DR for T2DM patients. It demonstrated relatively good discrimination and calibration power, which identified that this nomogram could be widely and accurately used for its large sample size [19]. Also, it still needs more effort to improve the model and make it more accurate and practical.

Age and course of disease are unmodifiable risk factors for T2DM patients. The result showed that younger age and longer course of disease were strongly connected with the incidence of DR. They should be as a pair of risk factors when we consider the association in it. Once T2DM is diagnosed, abnormally elevated blood sugar induces oxidative stress and causes microinflammation [20] which is considered the important pathogenesis of T2DM and its complication [21–23]. With age growing and microinflammation developing continuously, the risk of DR increases undoubtedly. Here, the risk factor of younger age may be about the element of social and era development. Younger age means earlier age of onset of T2DM. Compared with those who are older-aged, those who are younger-aged were born and have been living in a more abundant era which meets the need of humans greatly while causes some effects in the human behavior and lifestyle meanwhile. For example, various kinds of delicious food satisfy people's taste buds but unhealthy eating habits would bring about obesity problem and T2DM further [24–26]. And with the development and changes of all aspects of society, people's life has become increasingly convenient and comfortable so that they may spend more time in front of the computers or in the office, leading to lack of time in experience to burn the calories and keep fit. So usually, the younger ages have got T2DM at their early age and suffered from the disease for a longer course, while those who are older-aged, with a later age of onset and shorter course of disease, have a lower risk of DR. From this point, the course of disease may be a more vital factor to the risk of DR.

Hyperglycemia is a well-known factor closely associated with DR development. Our study also indicates that higher PBG and HbA1c may contribute to the risk of DR. One study reported that by categorizing PBG and HbA1c by deciles, with the prevalence of DR calculated in each decile, the prevalence of DR increased sharply in the 10th decile, when HbA1c exceeded 6.4%. The threshold of HbA1c for detecting DR is nearly consistent with the criteria for diagnosing diabetes from the World Health Organization [27], meaning that once T2DM is diagnosed, there is already DR complication or a high probability. Another showed that every 1% increase in HbA1c increased the risk of DR by 1.53 times, and every 1 mmol/L increase in PBG increased the risk of DR by 1.07 times [28]. And more researches have proved the positive correlation between DR and hyperglycemia [29, 30]. The UK Prospective Diabetes Study, one of the landmark clinical trials, suggested tight glycemic control could reduce the risk of DR development and progression in T2DM patients [31].

As we all know, UMA and UCR are biochemical indicators reflecting renal function, and T2DM is a kind of chronic disease with systemic metabolic disorders. So the abnormal renal function metabolic indicators not only can indicate the renal disease but also suggest the risk of indirectly associated lesions like eye disease. Previous studies have proved that UMA is a strong predictor for DR and hard exudate formation in type 2 diabetics even after correcting for the duration of diabetes and other systemic risk factors [32]. Both glomerular filtration rate and the ratio of UMA to UCR are important risk factors for DR, and the ratio of UMA to UCR has a better association with DR [33], which is consistent with the result of our study, even, there is a report suggesting that microalbuminuria is the tip of the iceberg of diabetic complications. It discovered that chronic complications of diabetes like diabetic retinopathy and neuropathy and ischemic heart disease were more common in the microalbuminuric group as compared to the nonmicroalbuminuric group [34]. Hence, UMA should be a valuable factor to predict diabetic complications.

The relationship between DR and hypertension has been suggested in some previous studies [35–37]. Our study also indicated that blood pressure may contribute to the risk of DR, especially high SBP [38–40]. For every 10 mmHg decrease in SBP, there was a reduction in 35% of DR, 35% need for retinal laser, and 50% blindness [41]. A large population-based cross-sectional study in the rage of Asia also showed that treated but poorly controlled as well as untreated hypertension was significantly associated with any DR. Among the blood pressure components, higher SBP and pulse pressure levels were associated with both any DR and vision-threatening DR [42].

T2DM is a kind of chronic lifelong disease, so it is extremely important to control disease development and prevent the occurrence of associated complications. Controlling blood glucose is always the first measure to decrease the risk of associated complications. However, most of the T2DM patients cannot achieve well-control blood glucose due to the lack of preventive consciousness or the lack of knowledge about T2DM, which indicates the increasing risk of associated complications. Therefore, it is meaningful to develop risk prediction tools to help doctors and T2DM patients raise vigilance and take preventive measures. DR is one of the diabetic complications with high incidence and great damage to health. So we developed a valid prediction tool, which assisted clinicians with early identification of patients at high risk of DR through the developed nomogram. Moreover, early interventions such as changing therapeutic scheme will benefit to decrease the risk of DR.

## 5. Limitations

There are some limitations to this study. Firstly, this study is based on the epidemiological data from a screening on community T2DM and its complication, so that the diagnosis of DR may lack strictness. So fundus photography with larger vision is needed and beneficial to ensure the accuracy of DR diagnosis. Secondly, that the predictive model showed medium prediction accuracy may suggest that more other indicators should be included such as inflammation-related indicators and oxidative stress-related indicators based on the mechanism of inflammation and oxidative stress [43, 44] and eye indicators related to retinopathy like information from optical coherence tomography and optical coherence tomography angiography [45]. Thirdly, though the population was relatively large, these communities mainly distributed on both banks of the Huangpu River, so enlarging the range of population would be better to increase the representation.

## 6. Conclusions

Combining with age, course of disease, PBG, HbA1c, UCR, UMA, and SBP, this study built a novel nomogram with relatively good accuracy to help clinicians and T2DM patients estimate the risk of DR. According to the evaluation result, clinicians and patients can take more targeted measures on medical interventions in time. As for the limitation of the predictive model, there is still a large space to improve the nomogram and increase the clinical utility.

## Figures and Tables

**Table 1:** Differences in characteristics between the training set and the validation set.

Variables	Total (*n* = 4170)	Training set (*n* = 3130)	Validation set (*n* = 1040)	*P* value
Age (years)	64.52 ± 6.75 (26, 88)	64.62 ± 6.77 (26, 88)	64.23 ± 6.70 (28, 85)	0.106
T2DM duration (years)	9.67 ± 6.85 (1, 61)	9.69 ± 6.83 (1, 59)	9.59 ± 6.90 (1, 61)	0.665
Gender				0.102
Female	2412 (57.8%)	1833 (58.6%)	579 (55.7%)	
Male	1758 (42.2%)	1297 (41.4%)	461 (44.3%)	
FBG (mmol/L)	7.98 ± 2.56 (3.9, 20.9)	7.97 ± 2.56 (3.9, 20.9)	8.02 ± 2.57 (4.4, 19.6)	0.552
PBG (mmol/L)	12.31 ± 4.75 (6.0, 30.0)	12.28 ± 4.76 (6.0, 30.0)	12.40 ± 4.74 (6.2, 29.1)	0.481
HbA1c (%)	7.29 ± 1.41 (4.6, 13.0)	7.30 ± 1.42 (4.6, 13.0)	7.27 ± 1.38 (4.6, 12.6)	0.652
TC (mmol/L)	4.89 ± 1.08 (2.07, 9.12)	4.89 ± 1.09 (2.07, 9.12)	4.88 ± 1.07 (2.26, 9.02)	0.681
TG (mmol/L)	1.89 ± 1.15 (0.45, 12.33)	1.91 ± 1.17 (0.45, 12.33)	1.85 ± 1.09 (0.56, 12.15)	0.202
LDL (mmol/L)	1.62 ± 0.46 (0.48, 3.20)	1.62 ± 0.46 (0.49, 3.20)	1.62 ± 0.45 (0.48, 3.19)	0.729
HDL (mmol/L)	1.57 ± 0.38 (0.54, 3.10)	1.57 ± 0.38 (0.54, 3.10)	1.57 ± 0.37 (0.57, 3.09)	0.770
SCR (*μ*mol/L)	67.49 ± 21.18 (25, 494)	67.34 ± 20.22 (25, 378)	67.96 ± 23.83 (30, 494)	0.412
BUN (mmol/L)	5.57 ± 1.63 (1.80, 18.84)	5.55 ± 1.63 (1.80, 18.84)	5.63 ± 1.62 (1.85, 14.28)	0.217
UA (*μ*mol/L)	314.05 ± 78.34 (97, 639)	313.91 ± 78.88 (97, 639)	314.48 ± 76.74 (123, 629)	0.837
UCR (mmol/L)	9.29 ± 4.19 (0.53, 18.44)	9.23 ± 4.17 (0.53, 18.44)	9.47 ± 4.24 (0.80, 18.33)	0.118
UMA (mg/L)	52.63 ± 74.22 (0, 270)	53.95 ± 75.84 (0, 270)	48.68 ± 68.98 (0, 270)	0.038
SBP (mmHg)	145.10 ± 19.32 (90, 210)	145.45 ± 19.42 (90, 210)	144.03 ± 18.99 (96, 209)	0.041
DBP (mmHg)	81.15 ± 10.50 (50, 120)	81.19 ± 10.41 (50, 120)	81.03 ± 10.77 (51, 118)	0.661
BMI (kg/m^2^)	25.57 ± 3.36 (16.38, 39.45)	25.58 ± 3.33 (16.38, 39.45)	25.54 ± 3.45 (16.53, 39.04)	0.729
WHR	0.91 ± 0.06 (0.69, 1.19)	0.91 ± 0.06 (0.69, 1.19)	0.91 ± 0.06 (0.69, 1.17)	0.682

**Table 2:** Predictors for the risk of DR in T2DM patients.

Intercept and variables	Prediction model					
	*β*	*z* value	*P* value	Odds ratio	CI (2.5%)	CI (97.5%)
Intercept	-3.037	-5.097	<0.001	0.048	0.015	0.154
Age	-0.031	-4.442	<0.001	0.969	0.956	0.983
Course	0.050	7.416	<0.001	1.051	1.038	1.066
PBG	0.027	2.349	0.019	1.027	1.004	1.050
HbA1c	0.258	7.058	<0.001	1.294	1.205	1.390
UMA	0.002	3.256	0.001	1.002	1.001	0.995
UCR	-0.027	-2.415	0.016	0.973	0.951	1.003
SBP	0.007	2.959	0.003	1.007	1.002	1.012

## Data Availability

The data used to support the findings of this study are available from the corresponding author upon request.
